# Evaluation of the ^129^I Half-Life Value Through Analyses of Primitive Meteorites

**DOI:** 10.7566/JPSCP.14.011005

**Published:** 2016-08-10

**Authors:** Olga PRAVDIVTSEVA, Alex MESHIK, Charles M. HOHENBERG

**Affiliations:** Department of Physics, CB 1105, Washington University, Saint Louis, MO 63130, USA

**Keywords:** ^129^I, half-life, primitive meteorites, I-Xe chronology, Pb-Pb chronology

## Abstract

The preserved record of decay of now-extinct ^129^I into ^129^Xe forms the basis of the I-Xe chronometer. Comparison of the high precision I-Xe and Pb-Pb ages of chondrules and pure mineral phases separated from eight meteorites suggests the 17.5 ÷ 14.6 Ma range for the ^129^I half-life, assuming that the ^235^U and ^238^U half-lives are correct. The mean value of 16 Ma indicates that the 15.7 Ma half-life of ^129^I used here for the I-Xe age calculations is most probably correct. Since the ^129^I half-life value only affects the relative I-Xe ages, the few Ma relative to the Shallowater standard, the absolute I-Xe ages are almost immune to this uncertainty in the ^129^I half-life.

## 1. Introduction

There are four experimental values available for the ^129^I half-life [Table I]. The recommended ^129^I half-life of 17 ± 1 Ma was derived from them as an unweighted average [5] and later as a weighted average, 16.1 ± 0.7 Ma [6], when experimental details for the more precise measurements [2, 3] became available. The half-life value of 15.7 ± 0.6 Ma [3] is also routinely used in the literature.

Here we independently evaluate the ^129^I half-life using the precise I-Xe ages of chondrules and different mineral phases separated from the primitive meteorites.

## 2. Experimental

### 2.1 I-Xe chronometry

Discovery of nucleosynthetic input into the Solar System within 10^7^–10^8^ years of its formation was made based on observations of an excess of stable ^129^Xe in a meteorite due to β decay of now-extinct ^129^I [7]. The preserved record of the ^129^I decay forms the basis of I-Xe radiometric dating, capable of deciphering the early Solar System processes with a high degree of precision [8].

In I-Xe dating the ^129^I/^127^I ratio at closure of the iodine host mineral is the value of interest. A tracer for ^127^I is iodine-derived ^*128^Xe, produced by neutron capture on stable ^127^I in a reactor

I127(n,γβ)X∗128e,X∗128e=I127·Φ·σ,

where Φ is neutron fluence, σ - effective cross section.

It is measured along with radiogenic ^*129^Xe produced by ^129^I decay, so the ^*129^Xe/^*128^Xe ratio provides the chronometry, avoiding the problems associated with measurements of absolute quantities of either parent, typically at 10–100 ppb level, or daughter. The direct monitoring of the neutron capture probability in ^127^I is avoided by use of a meteoritic standard, which is irradiated along with the sample. The I-Xe age Δt of the sample is then determined relative to the standard [8]:

(X∗129eX∗128e)0=(X∗129eX∗128e)0standard·e-Δt/τ,

where τ is the mean lifetime of ^129^I.

### 2.2 Comparison of the I-Xe and Pb-Pb ages

**Fig. 1:**
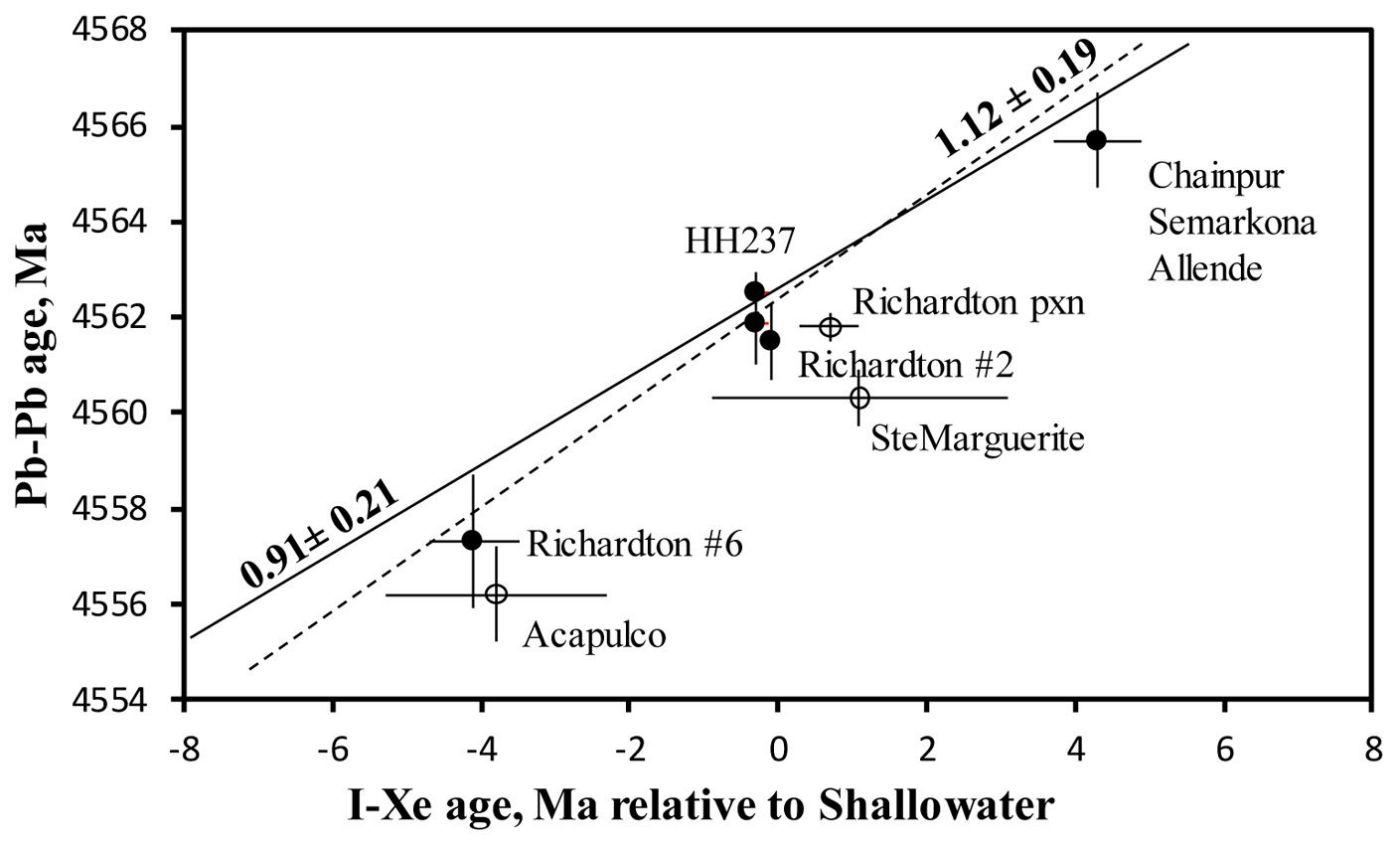
Comparison of I-Xe and U-corrected P-Pb ages measured in a range of materials from the early Solar System [Table II]. The dotted free-fit correlation line is based on all available data. The solid free fit correlation line is based on chondrules data only (solid symbols). Both are plotted using a weighted total least-squares algorithm. All I-Xe ages are calculated assuming the ^129^I half-life of 15.7 Ma.

When two chronometers experience concordant evolution and all the decay constants are correct, the corresponding ages, measured in the same samples, fall on a correlation line with the slope of 1 (Fig. 1). For the evaluation of the ^129^I half-life value, I-Xe ages should be compared to a high precision chronometer, Pb-Pb being a logical choice, with the implicit assumption that both chronometers closed at the same time and date the same event. Unfortunately, minerals rich in iodine (hence radiogenic ^*129^Xe) usually have concentrations of uranium that are too low for the high precision Pb-Pb dating, and *vice versa*. Very few meteoritic materials fit the requirements for both good Pb-Pb and good I-Xe ages.

Samples used for the comparison of I-Xe and Pb-Pb ages are combined in Table II. I-Xe ages are calculated using the ^129^I half-life of 15.7 Ma. The Pb-Pb ages are corrected using the latest ^238^U/^235^U ratio for the Earth and the Solar System of 137.794 ± 0.027 and meteorite specific ^238^U/^235^U ratios for Richardton (137.711 ± 0.008) and Acapulco (137.796 ± 0.013) [10]. An average of two Allende bulk ^238^U/^235^U ratios [10] was applied for the correction of the Pb-Pb ages of the earliest chondrules. The ^238^U/^235^U ratio of 137.794 ± 0.014 for Gujba [9] was used here as a proxy for the HH 237 ^238^U/^235^U ratio value.

The slopes of the I-Xe – Pb-Pb correlation lines range from 1.12 ± 0.19 for the whole set of data points from Table II to 0.91 ± 0.21, when only chondrule data are considered [solid symbols, Fig. 1]. In order to bring the slope values to 1, the half-life of ^129^I should be ~17.4 Ma when all data are considered, or ~ 14.6 Ma for the chondrules- only correlation.

Additional data points (not listed in Table II) consistently bring the slope of the regression bellow 1.12. However, the underlying problem here is the divergence of minerals suitable for dating by both chronometers. In fact, HH237 chondrule is the only sample in this compilation with strong isotopic and mineralogical evidence for the simultaneous closure of the I-Xe and Pb-Pb systems [14], in line with the proposed formation of CB chondrules from a melt fraction of the impact-generated plume [15].

## 3. Conclusion

Comparison of precise I-Xe and Pb-Pb ages measured in chondrules and mineral phases separated from eight meteorites suggests the 17.5 ÷ 14.6 Ma range for the ^129^I half-life value, assuming that the ^235^U and ^238^U half-lives are correct. The mean value of 16 Ma indicates that the 15.7 Ma half-life of ^129^I used here for the I-Xe age calculations is most probably correct. Since the ^129^I half-life only affects the relative I-Xe ages, the absolute I-Xe ages, calculated relative to the Shallowater standard age of 4562.4 ± 0.2 Ma, are almost immune to this uncertainty in the ^129^I half-life.

## Figures and Tables

**Table I:** The experimental half-life values of ^129^I.

Author (year of publication)	T_1/2_ Ma
[1] Katcoff (1951)	17.2 ± 0.9
[2] Russel (1957)	15.6 ± 0.6
[3] Emery (1972)	15.7 ± 0.6
[4] Kuhry (1973)	19.7 ± 1.4

**Table II:** Samples used for the I-Xe Pb-Pb ages correlation plots and their respective ages. I-Xe ages are relative to the meteoritic standard Shallowater.

Meteorite (Type)	I-Xe age, 10^6^ years Shallowater ≡ 0		Pb-Pb age, 10^6^ years	
Chainpur, LL3.4 Semarkona, LL 3.0 Allende, CV3	chondrules [11, 12]	4.3 ± 0.6	chondrules [13]	4565.6 ± 1.0
HH 237 (CB)	chondrule [14]	−0.29 ± 0.16	silicates [15]	4561.9 ± 0.9
			Gujba chondrules[16]	4562.5 ± 0.2
Richardton (H4)	chondrule 2 [17]	−0.1 ± 0.1	chondrule 2 [17]	4561.5 ± 0.8
	chondrule 6 [17]	−4.1 ± 0.6	chondrule 6 [17]	4557.3 ± 1.4
	pyroxene [18]	1.1 ± 2.0	pyroxene [19]	4560.3 ± 0.6
Acapulco	feldspar [20]	−3.8 ± 1.5	phosphate [21]	4556.2 ± 1.0
Ste Marguerite(H4)	feldspar [20]	0.7 ± 0.4	phosphate [21]	4561.8 ± 0.3
